# Multifunctional Lactoferrin Coatings on PLA/HAp Microspheres for Antibacterial, Pro-Adhesive and Osteogenic Effect on MC3T3-E1 Cells

**DOI:** 10.3390/ijms27167069

**Published:** 2026-08-07

**Authors:** Bartosz Mielan, Magdalena Pajączkowska, Joanna Nowicka, Marcel Zambrzycki

**Affiliations:** 1 Pre-Clinical Research Centre, Wroclaw Medical University, Marcinkowskiego 1, 50-368 Wroclaw, Poland; 2 Department of Microbiology, Faculty of Medicine, Wroclaw Medical University, Chałubinskiego 4, 50-368 Wroclaw, Poland; 3 Department of Biomaterials and Composites, Faculty of Materials Science and Technology, AGH University of Kraków, Mickiewicza 30, 30-059 Kraków, Poland

**Keywords:** antimicrobial activity, cell adhesion, cell carriers, lactoferrin, microspheres, modular tissue engineering, osteogenesis, poly(L-lactic acid) (PLA), surface engineering

## Abstract

Modern biomaterials are increasingly expected to fulfill multiple functions simultaneously. The aim of this study was to develop multifunctional composite microspheres (MSs) based on poly(L-lactic acid) and hydroxyapatite (PLA/HAp), surface-modified with lactoferrin (Lf). The objective was to create a material exhibiting antimicrobial, pro-adhesive, and osteogenic properties for potential applications in modular tissue engineering. Microspheres with diameters ranging from 70 to 150 µm were fabricated using an oil-in-water emulsification method and subsequently coated with lactoferrin. Antimicrobial efficacy was evaluated against *S. aureus*, *P. aeruginosa*, and *C. albicans* by assessing bacterial adhesion, colony formation, and reduction rates. Osteoblast behavior, including adhesion and differentiation, was investigated using the MC3T3 cell line. The morphology of the samples was analyzed via optical and scanning electron microscopy. The results demonstrated that Lf-coated MSs exerted a significant antibacterial effect against *S. aureus* and *P. aeruginosa*. Furthermore, the presence of lactoferrin enhanced cell adhesion to the microspheres, as confirmed by the MTT assay after 24 h of incubation. On day 14, osteogenic differentiation was observed in cultures containing Lf-coated MSs, even in the absence of exogenous differentiating factors. Additionally, hematoxylin and eosin (H/E) staining revealed improved self-assembly of the Lf-modified microspheres. The reduction in microbial adhesion, coupled with enhanced osteoblast attachment, suggests a synergistic effect that may increase the success rate of implant integration at an early stage.

## 1. Introduction

Lactoferrin (Lf) is a promising alternative iron-binding cationic glycoprotein with diverse biomedical applications. It can be incorporated into biomaterials to induce antibacterial properties, promote osteogenesis, and serve as an effective agent for surface modification [1,2,3]. Beyond its antimicrobial activity, Lf exhibits antioxidative, immunomodulatory, anti-inflammatory, and even anti-cancer properties. Its antimicrobial mechanism is primarily attributed to its ability to sequester iron ions essential for bacterial growth—a process further enhanced by the acidic environment typical of inflammation sites, such as the accumulation of lactic acid produced by bacteria [4,5,6] or resulting from the degradation of poly(lactic acid). Some researchers also propose an additional antibacterial mechanism based on the presence of large cationic patches on the Lf molecule, which may simultaneously contribute to the acceleration of wound healing [7]. The antibacterial efficacy of Lf-loaded hydrogels against *S. aureus* and *P. aeruginosa* has been well-documented, showing significant reductions in bacterial viability and colony growth area [8]. The synergy of Lf’s properties—namely its positive charge, the presence of surface hydrophobic domains, affinity for bacterial surfaces, and iron-binding capacity—makes it an excellent candidate for the development of antibacterial materials [9,10]. Furthermore, Lf can bind to various substances, including lipopolysaccharides, glycosaminoglycans, and metal ions such as Zn^2+^, Cu^2+^, and Al^3+^ [11], which opens new possibilities for molecular modification and metal ion sequestration.

Yamada et al. demonstrated that Lf exerts anti-osteoclastogenic effects. It achieves this by inhibiting the interaction between osteoblasts and osteoclast precursors, and by directly suppressing osteoclast formation and maturation through the blockade of the RANKL-RANK signaling pathway [12] and the downregulation of mRNA expression associated with calcitonin receptor synthesis [13]. This activity represents a critical factor in bone regeneration and remodeling processes. Furthermore, Lf acts as an inhibitor of osteoblast apoptosis, demonstrating efficacy even at low concentrations [14]. As an anabolic factor, Lf strongly promotes bone growth; although oral supplementation has been shown to increase bone mineral density in vivo [13], local delivery is preferred to mitigate potential systemic side effects. Chang et al. confirmed that the presence of Lf enhances the expression of osteogenic factors in various cell lines [15]. Specifically, the proliferation and osteogenic differentiation of MC3T3 cells can be significantly improved by Lf supplementation [13,16]. Ultimately, Lf shifts the homeostatic balance between osteoblasts and osteoclasts towards osteogenesis [17].

The surface of a biomaterial is the primary determinant of its biocompatibility and plays a significant role in cell adhesion. Numerous methods exist to modify material surfaces, thereby influencing their chemical composition, functional groups, microstructure (e.g., porosity and pore size), topography (e.g., roughness), and their physical (wettability, electrical, and magnetic) or mechanical properties [18,19,20].

Cell adhesion is a critical process in tissue engineering and regenerative medicine. The initial interaction between a biomaterial and the biological environment occurs in a series of stages, starting with water adsorption, followed by protein adsorption and, subsequently, cell attachment—all of which are essential for successful implantation. Adsorbed proteins create specific adhesion sites that facilitate cell integration. Consequently, surface modification of biomaterials with bioactive peptides (e.g., the RGD sequence, consisting of arginine, glycine, and aspartic acid) is frequently employed to promote cell adhesion [21,22]. Proteins coating the surface interact with cells through electrostatic, physical, and chemical mechanisms [23]. Cell adhesion to these specific proteins or amino acid sequences triggers signaling pathways involving surface receptors and integrins, which in turn induce rearrangements in the cytoskeleton. These processes ultimately influence cell migration, adhesion, intercellular communication, proliferation, and differentiation—including osteogenic differentiation, which is essential for effective bone tissue regeneration [18,19,24,25].

Unfortunately, although strategies to improve host cell adhesion are well-established, various microorganisms present in the body and the environment are also attracted to implant surfaces. The initial interaction between an implant and the biological environment is characterized by a competition between host cells and microorganisms, a phenomenon commonly referred to as the “race for the surface” [26], which ultimately determines the success or failure of the implantation. Bacteria colonize surfaces non-specifically through multiple physical interactions, including van der Waals forces, electrostatic interactions, Brownian motion, diffusion, and active motility [27,28]. Furthermore, the bacterial growth rate is significantly higher than that of eukaryotic cells due to a much shorter doubling time (approximately 20 min versus 6–24 h, respectively) [29]. For this reason, developing strategies to prevent bacterial adhesion and proliferation on implant surfaces is of paramount importance.

*Staphylococcus aureus* biofilm is a primary factor contributing to implant failure and subsequent infection. Once established, the biofilm facilitates nutrient exchange and intercellular communication, creates a three-dimensional matrix for bacterial adhesion and growth, and provides a protective polysaccharide barrier that renders antibiotic therapy less effective [30,31]. Similar to host cells, bacterial adhesion to biomaterials can be modulated through various strategies, including the incorporation of antibiotics or antimicrobial agents, as well as the engineering of surface morphology [30].

Lf possesses potent antibacterial properties. Furthermore, the presence of RGD sequences within the Lf molecule promotes cell adhesion [32]. While Kim et al. utilized Lf-loaded microspheres (MSs) to induce cellular differentiation [33], other critical parameters—such as proliferation, adhesion, and the influence of the three-dimensional microstructure—remained unexamined. In this study, we analyze the interaction of Lf-coated 3D microspheres with both microorganisms and MC3T3 cells. By simultaneously enhancing osteoblast adhesion and exerting antimicrobial activity, Lf is expected to shift the balance of the “race for the surface” in favor of host cells, thereby improving the potential for successful clinical integration.

## 2. Results

### 2.1. Characterization of the Microspheres

**Figure 1:**
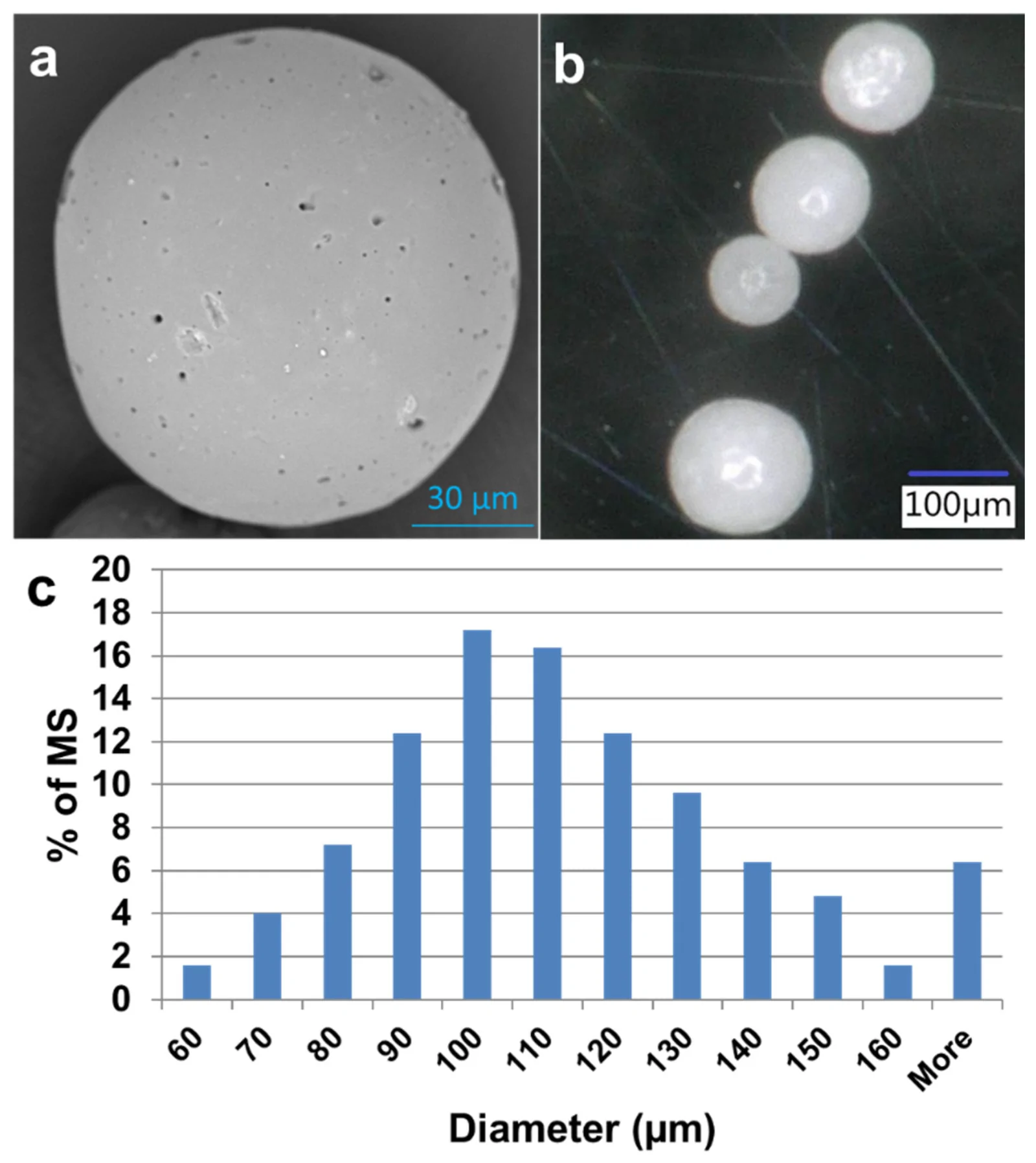
Morphology and size distribution of PLA/Hap microspheres. (**a**) Scanning electron microscopy (SEM) image illustrating the spherical geometry and surface topography of a representative microsphere, showing small, crater-like pores resulting from solvent evaporation. (**b**) Optical microscopy image showing the general appearance and size range of the microspheres. (**c**) Histogram representing the diameter distribution of the fabricated microspheres, indicating a mean diameter within the 70–150 µm range.

The morphology of the microspheres (MSs) is presented in Figure 1. The MSs exhibited a regular, spherical shape with a generally smooth surface, interspersed with occasional crater-like pores (up to 5 µm in diameter). These surface features likely resulted from the solvent evaporation process during synthesis.

The diameter distribution of the MSs was analyzed; although it visually resembled a normal distribution, the Shapiro–Wilk test rejected the null hypothesis of normality (*p* < 0.05). Approximately 90% of the MS population fell within the 70–150 µm diameter range, with a distinct mode of 100–110 µm (comprising over 30% of the samples). A small number of outliers with diameters exceeding 170 µm were observed. Additionally, a minor fraction of irregular structures, such as short fibers, was identified during the synthesis; these were largely removed manually prior to further testing.

### 2.2. Antibacterial Properties

#### 2.2.1. Quantitative Assessment of Microbial Adhesion

**Figure 2:**
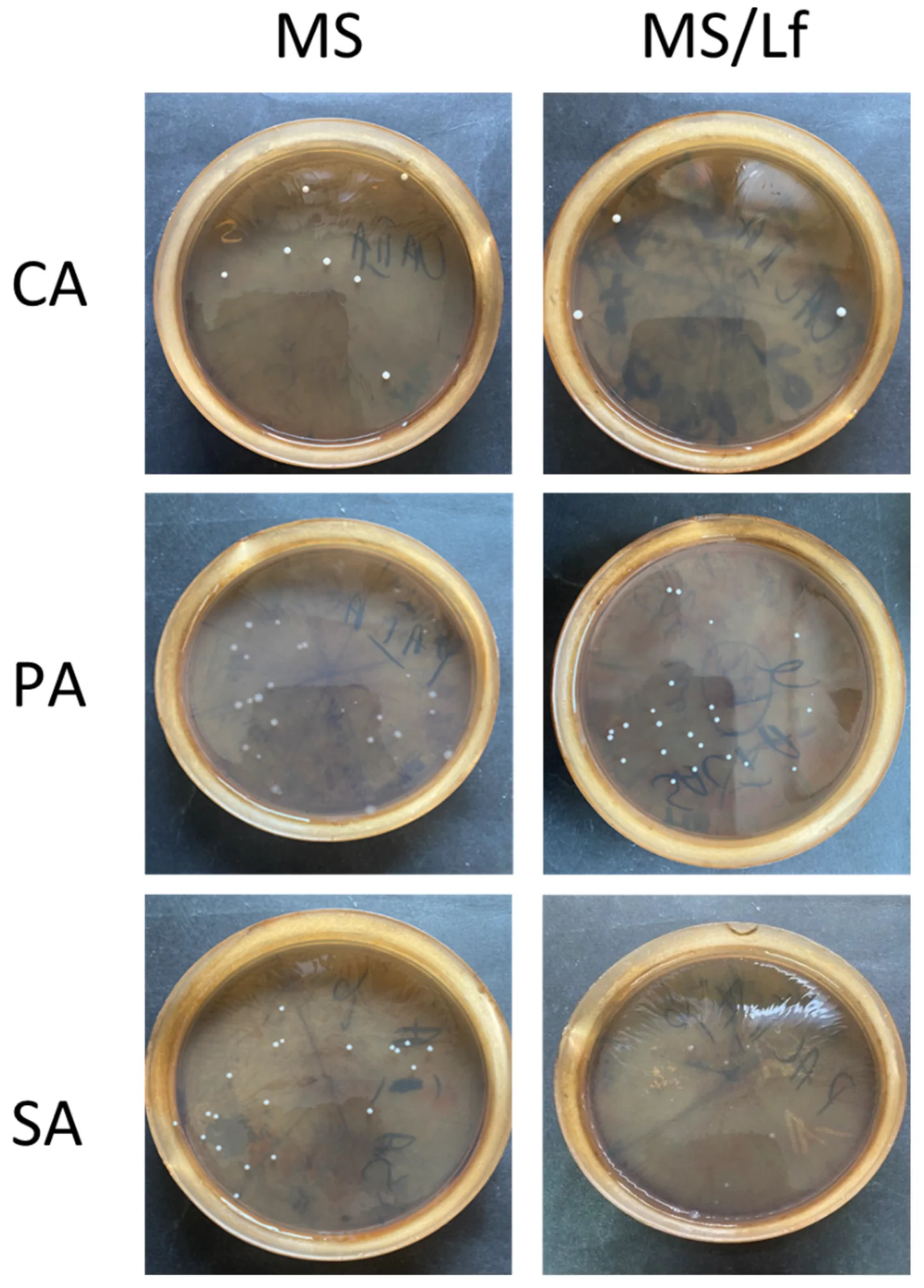
Quantitative analysis of microbial adhesion on PLA/HAp microspheres. Representative images of colonies formed on agar plates after incubation with MSs (**left** column) and Lf-coated MSs (**right** column) for: (CA) *Candida albicans*, (PA) *Pseudomonas aeruginosa*, and (SA) *Staphylococcus aureus*. The reduction in the number of colony-forming units is clearly visible for the Lf-modified samples across all tested strains, with the most significant inhibitory effect observed for *S. aureus*.

The quantitative results of microbial adhesion to the tested microspheres are presented in Figure 2. The data indicate that the presence of Lf on the surface of the microspheres effectively reduces the adhesion and colony-forming capacity of the investigated microorganisms (*S. aureus*, *P. aeruginosa*, and *C. albicans*).

Specifically, for *C. albicans*, the number of colonies observed on unmodified MSs was 7, whereas the Lf-coated MSs yielded only 3 colonies, representing a reduction of approximately 50%. In the case of *P. aeruginosa*, the control MSs showed 29 colonies, while the Lf-modified MSs showed 23 colonies, indicating a less pronounced inhibitory effect for this strain. The most significant antibacterial activity was observed against *S. aureus*; the number of colonies decreased from 20 on the unmodified MSs to 6 on the Lf-coated MSs, reflecting a nearly four-fold reduction in colony formation. These findings demonstrate that surface modification with Lf consistently reduces microbial adhesion across all tested strains, with the highest efficacy demonstrated against *S. aureus*.

**Figure 3:**
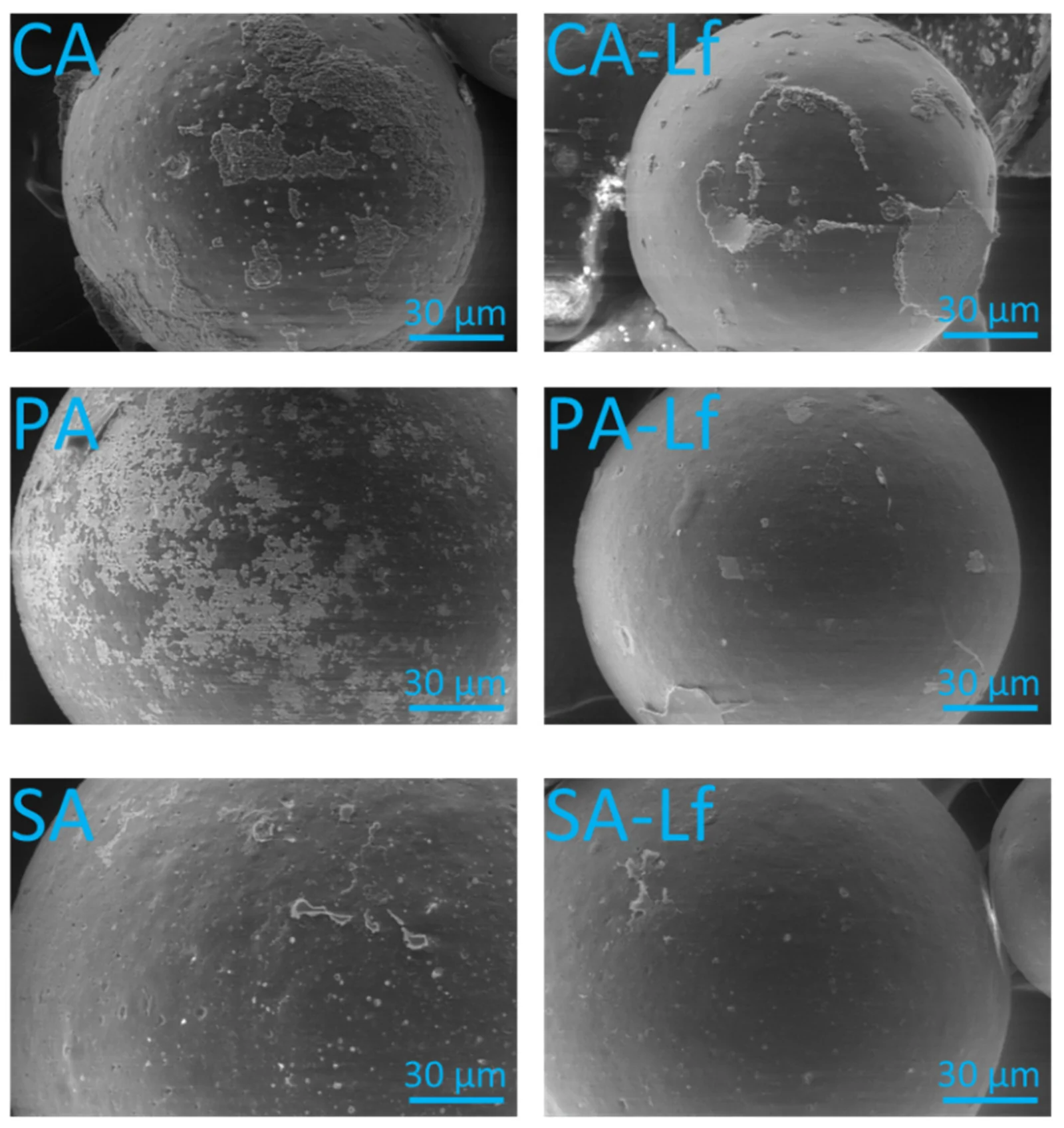
SEM images of microbial adhesion on microsphere surfaces. Representative SEM micrographs showing the colonization of unmodified microspheres (**left** column) and Lf-coated microspheres (**right** column) by *Candida albicans* (CA), *Pseudomonas aeruginosa* (PA), and *Staphylococcus aureus* (SA). A visible reduction in the density of adherent microbial cells is observed on the Lf-modified surfaces compared to the non-modified controls, confirming the inhibitory effect of lactoferrin coating on microbial adhesion and biofilm formation.

The SEM analysis presented in Figure 3 illustrates the microbial colonization of unmodified and Lf-coated (MS). The observations for the three tested microorganisms are as follows:

*Candida albicans* (*CA*): On unmodified MS, *C. albicans* forms large, flat layers that cover the majority of the material surface. On Lf-coated samples, the surface area covered by *C. albicans* is significantly reduced. *C. albicans* cells on the Lf-modified surface tend to form three-dimensional structures rather than spreading across the material, suggesting that the surface is less favorable for *C. albicans* growth.

*Pseudomonas aeruginosa* (*PA*): Unmodified MSs show extensive bacterial coverage, with cells forming colonies across the material. On Lf-coated MSs, the number of colonies is significantly lower. Similar to *C. albicans*, *P. aeruginosa* on Lf-modified surfaces prefers to form dense, interconnected structures rather than adhering directly to the material. Small sheet-like structures suggest an interconnection between cells and MS, though these are notably smaller than those observed for *C. albicans*.

*Staphylococcus aureus* (*SA*): On unmodified PLA/HAp material, *S. aureus* colonizes significantly smaller areas compared to *C. albicans* and *P. aeruginosa*, suggesting that the base material itself is less attractive to *S. aureus*. Minimal three-dimensional sheets of *S. aureus* are visible on the unmodified surface. Surface modification with Lf further decreases the number of colonies, and cell-to-cell interconnection is negligible.

**Figure 4:**
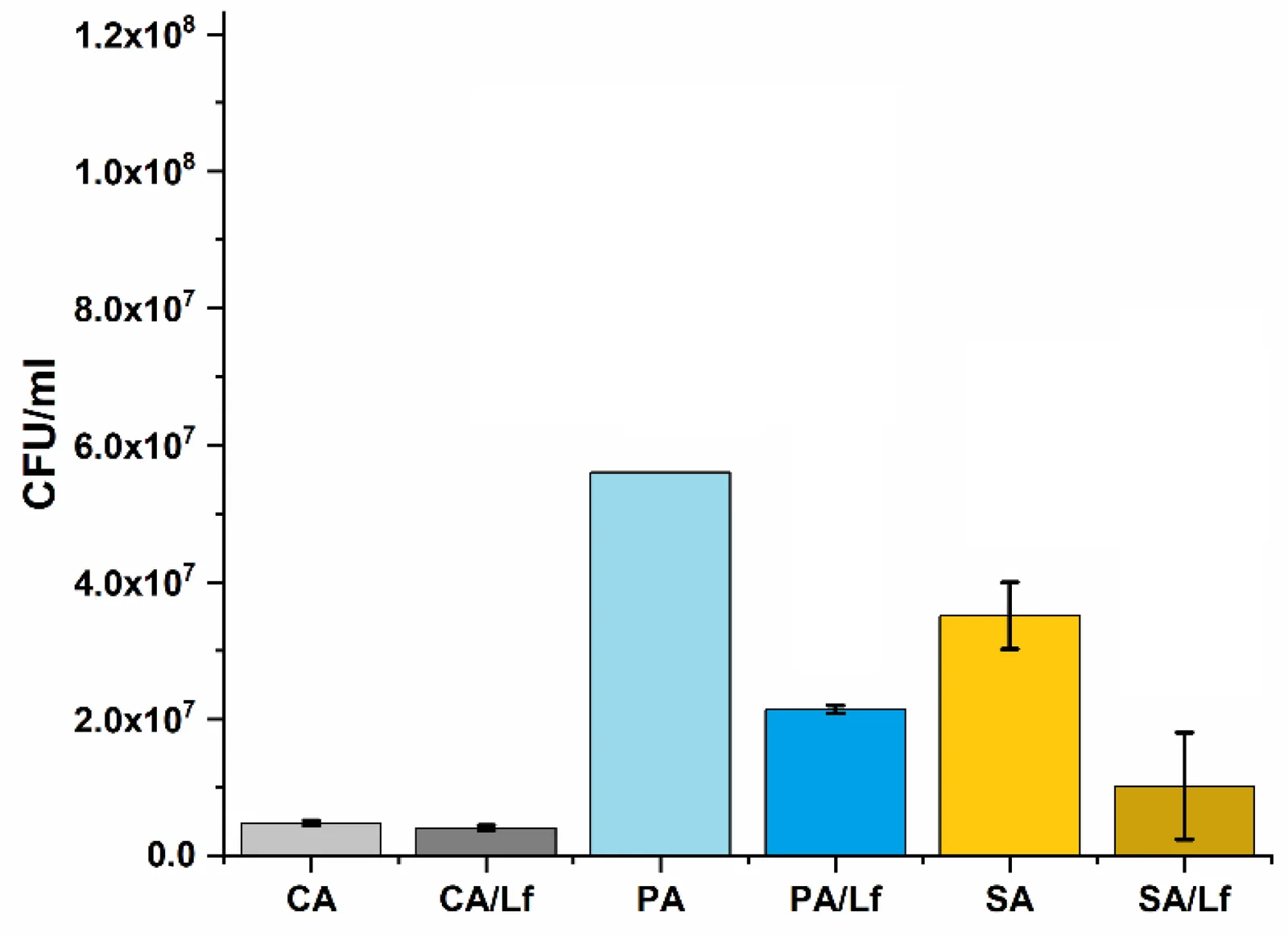
Adhesion level of microorganisms to the surface of MS; unmodified (-) and modified with lactoferrin (Lf). (CA—*C. albicans*; PA—*P. aeruginosa*; SA—*S. aureus*).

The adhesion levels of microorganisms to the MS surfaces—both unmodified (MS) and modified with lactoferrin (MS/Lf)—are presented in Figure 4. The results indicate that the Lf coating significantly influences the colonization of the material, depending on the microbial strain. For *C. albicans* (CA), no significant difference in adhesion was observed between unmodified MSs and MS/Lf. In the case of *P. aeruginosa* (PA), the presence of the Lf coating led to a substantial reduction in bacterial adhesion, with the CFU/mL value being approximately 2.6 times lower compared to the unmodified MS. The most pronounced inhibitory effect was observed for *S. aureus* (SA), where the Lf modification decreased the adhesion level by approximately 3.4 times relative to the control group.

These findings demonstrate that surface functionalization with Lf effectively reduces the adhesion of bacterial cells to the material, which may be a crucial factor in decreasing the risk of post-implantation infections.

#### 2.2.2. Antimicrobial Activity Against Planktonic Microbial Forms

**Figure 5:**
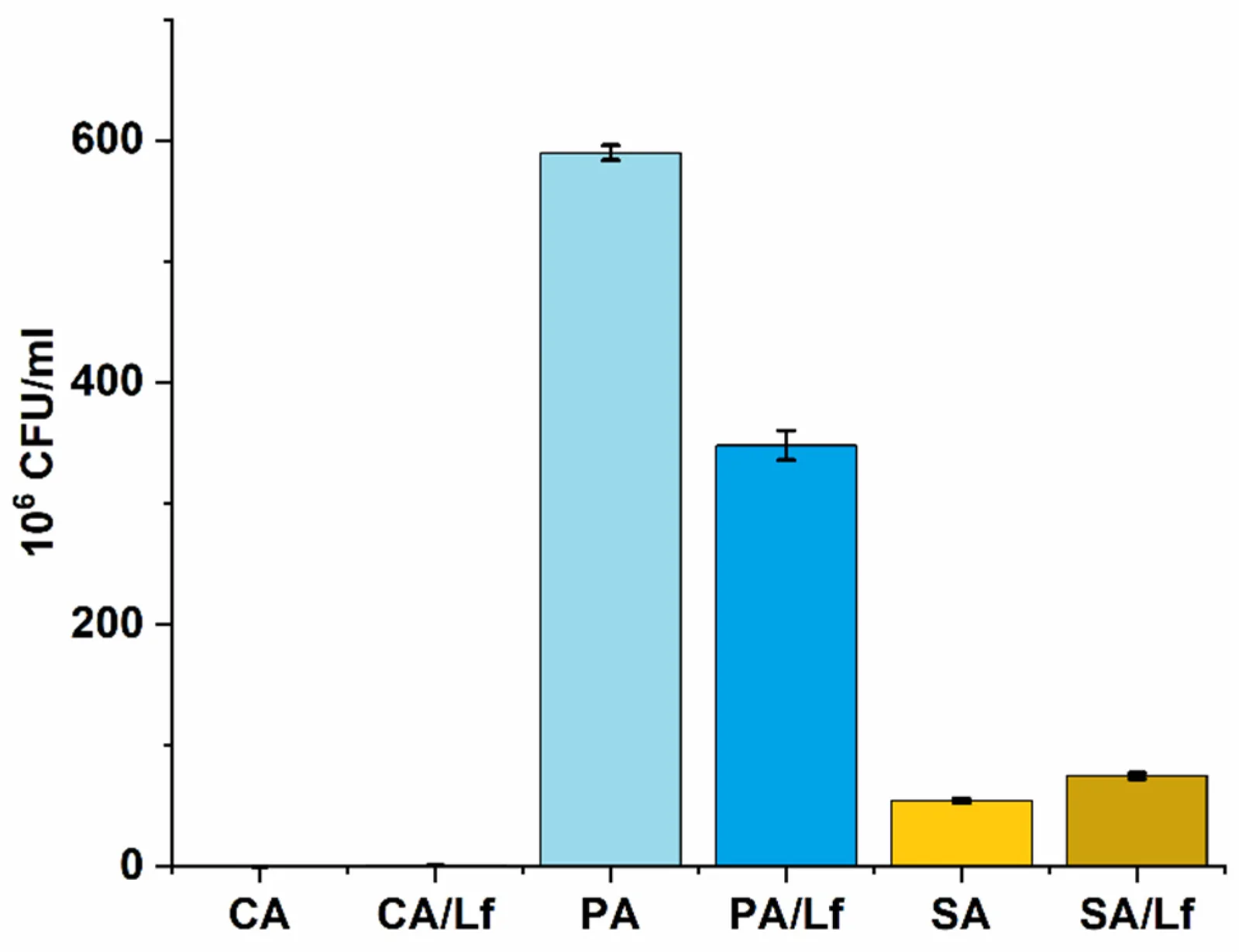
Value of colony-forming units per milliliter of suspension (CFU/mL).

The quantitative antimicrobial results for the planktonic forms of the selected microorganisms in contact with unmodified (MS) and Lf-coated microspheres (MS/Lf) are presented in Figure 5. The analysis of colony-forming units (CFU/mL) yields the following observations. For *Candida albicans*: The addition of Lf resulted in a minimal increase in CFU/mL compared to the unmodified MSs (8.5 × 10^5^ and 5.1 × 10^4,^ respectively), which may suggest that this condition favored fungal presence. Conversely, an inhibitory effect was recorded for *Pseudomonas aeruginosa*, where the CFU/mL value decreased from 5.8 × 10^8^ for unmodified MSs to 3.5 × 10^8^ for MS/Lf, representing a reduction of approximately 60%. Regarding *Staphylococcus aureus,* an increase in CFU/mL from 5.4 × 10^7^ to approximately 7.5 × 10^7^ was observed upon the addition of Lf, indicating no reduction in bacterial count under these planktonic test conditions.

**Figure 6:**
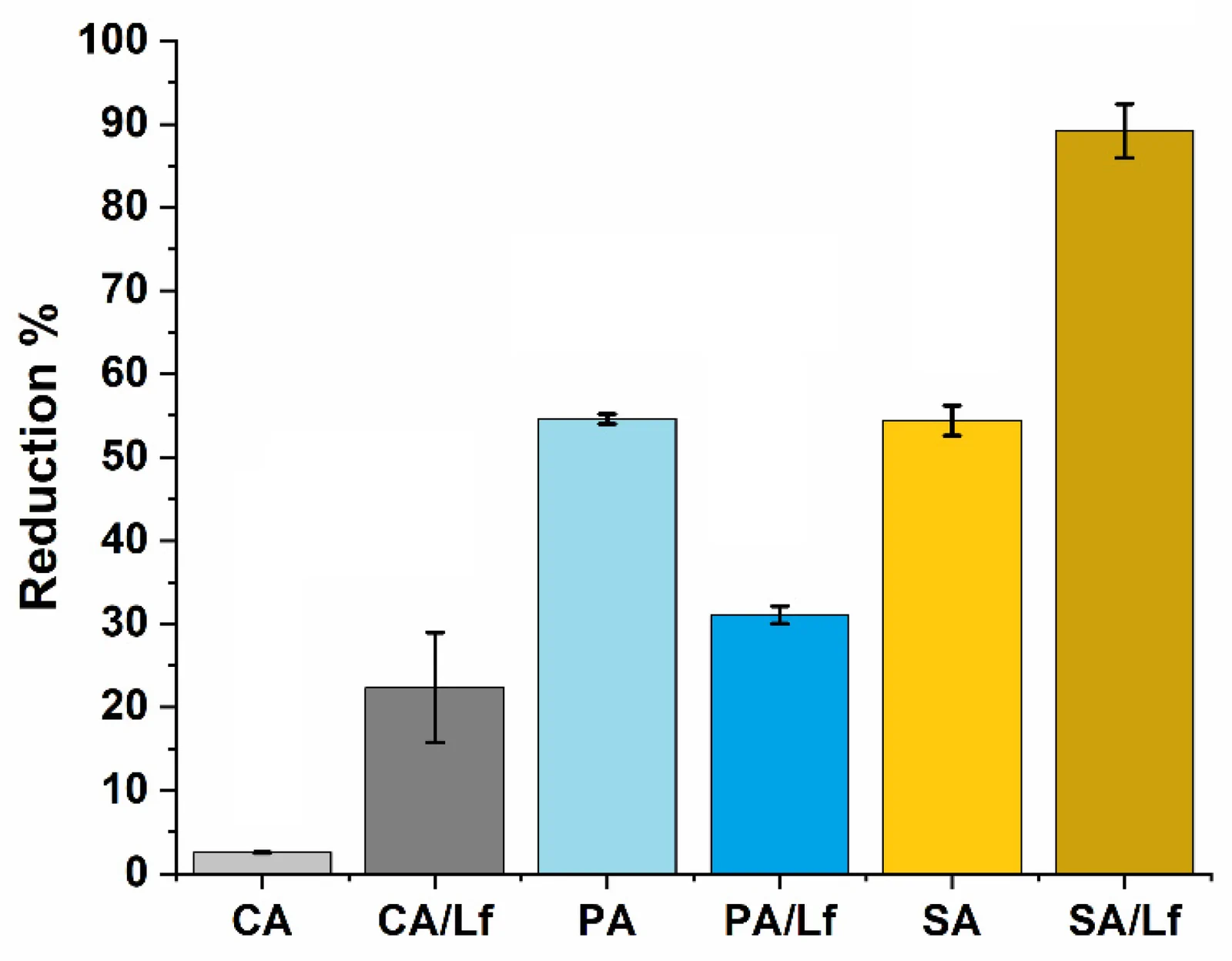
Reduction in microbial colonies in suspension in contact with MS without (-) and with lactoferrin (Lf).

Figure 6 illustrates the percentage reduction in microbial colonies in the presence of uncoated microspheres and Lf-coated microspheres, using a suspension of microorganisms without MSs as a reference. For *Candida albicans,* the presence of MSs resulted in a low reduction rate of approximately 5%, which significantly increased to around 20% with the addition of Lf. Uncoated MSs reduced the *Pseudomonas aeruginosa* colony count by approximately 55%, whereas MS/Lf showed a lower reduction rate of around 30%. This atypical trend, compared to the other tested microorganisms, may be attributed to the secretion of siderophores by *P. aeruginosa*, which can compete with Lf for iron affinity. The presence of MSs reduced *S. aureus* colonies by 55%, while modification with Lf increased this reduction to 90%, representing the highest reduction rate observed among all samples.

### 2.3. Microstructure of MSs with MC3T3 Cells at Day 7

**Figure 7:**
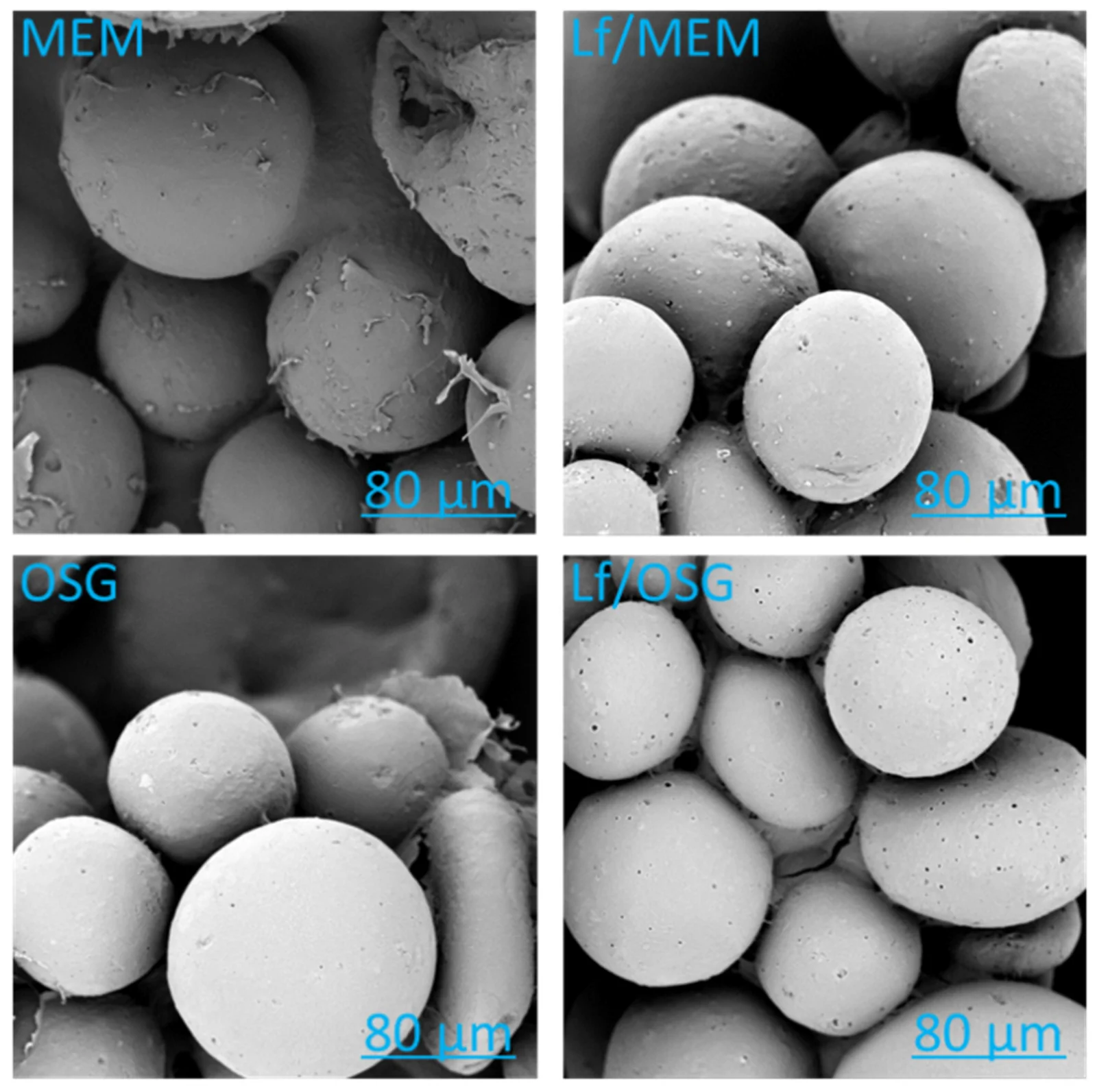
Microstructure of MSs after 7 days in contact with MC3T3 cells under SEM; MEM—MSs with minimal essential medium, MEM/Lf—MSs coated with Lf in minimal essential medium, OSG—MSs in OSG medium, OSG/Lf—MSs coated with Lf in OSG medium.

The microstructure of the microspheres (MSs) in contact with MC3T3-E1 cells at day 7 reveals a complex, self-assembled organization Figure 7. Although the microspheres were initially introduced in a separate form, by day 7 they had self-assembled into dense, three-dimensional, stacked structures. It appears that the cells actively rearranged the microspheres within the wells, forming 3D constructs according to their spatial preferences. These constructs are heavily covered with cells and extracellular matrix (ECM), with particularly dense coverage observed in the spaces between individual microspheres. The outer surfaces are also richly populated by cells, sometimes forming thin layers that appear slightly detached from the material.

**Figure 8:**
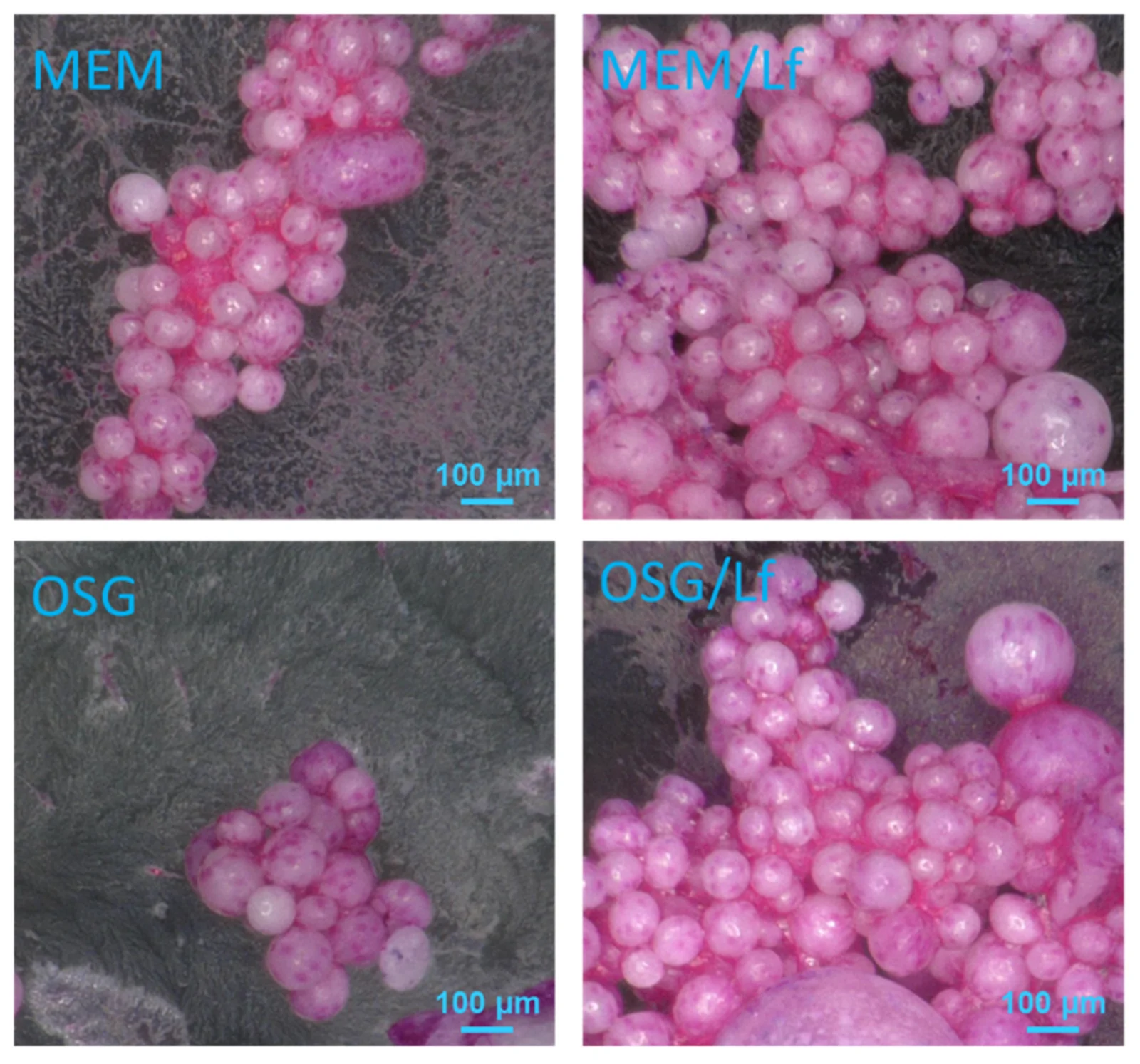
Microstructure of MSs after 7 days in contact with MC3T3 cells stained with hematoxin/eosin; MEM—MSs with minimal essential medium, MEM/Lf—MSs coated with Lf in minimal essential medium, OSG—MS in OSG medium, OSG/Lf—MSs coated with Lf in OSG medium.

While scanning electron microscopy (SEM) did not reveal significant differences between the samples, H/E staining provided further insight Figure 8. The surface of all materials was covered with cells, with the highest density observed in the interstitial spaces between individual MSs. Notably, the number of microspheres contained within these cellular agglomerates was significantly higher for MSs coated with Lf compared to the pure MS. This increased degree of agglomeration suggests that the presence of lactoferrin effectively promotes cell adhesion.

**Figure 9:**
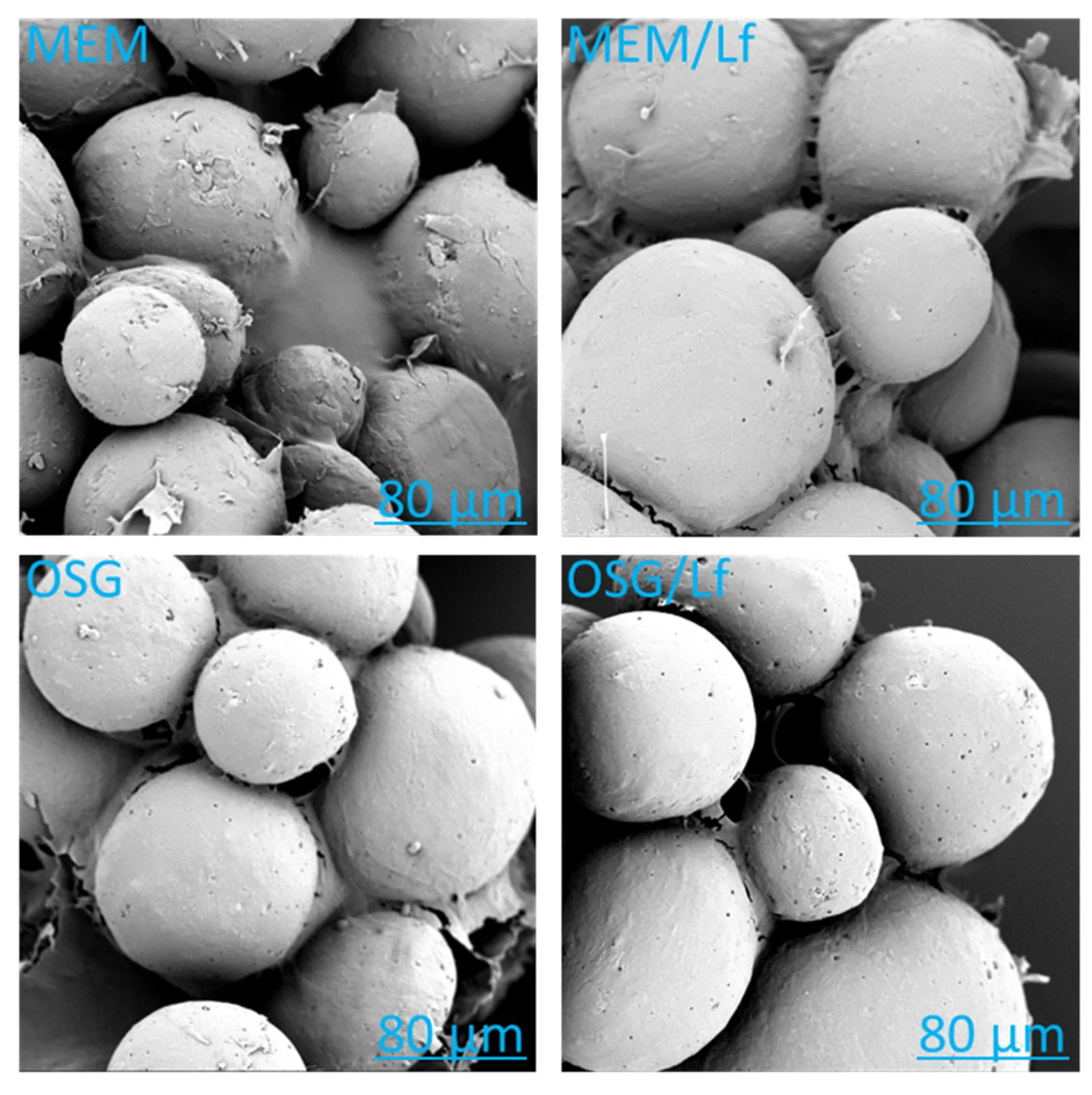
Microstructure of MSs after 14 days in contact with MC3T3 cells under SEM; MEM—MSs with minimal essential medium, MEM/Lf—MSs coated with Lf in minimal essential medium, OSG—MSs in OSG medium, OSG/Lf—MSs coated with Lf in OSG medium.

At day 14, SEM analysis Figure 9 reveals an increase in the amount of both ECM and cells compared to the observations made at day 7. The coverage extends beyond the center of the constructs to include smaller spaces between individual microspheres, where cells and ECM frequently appear as small sheets on the material surface or as strings bridging the microspheres. Consistent with the day 7 findings, no significant differences were observed between the samples regarding the presence of Lf or the type of culture medium.

**Figure 10:**
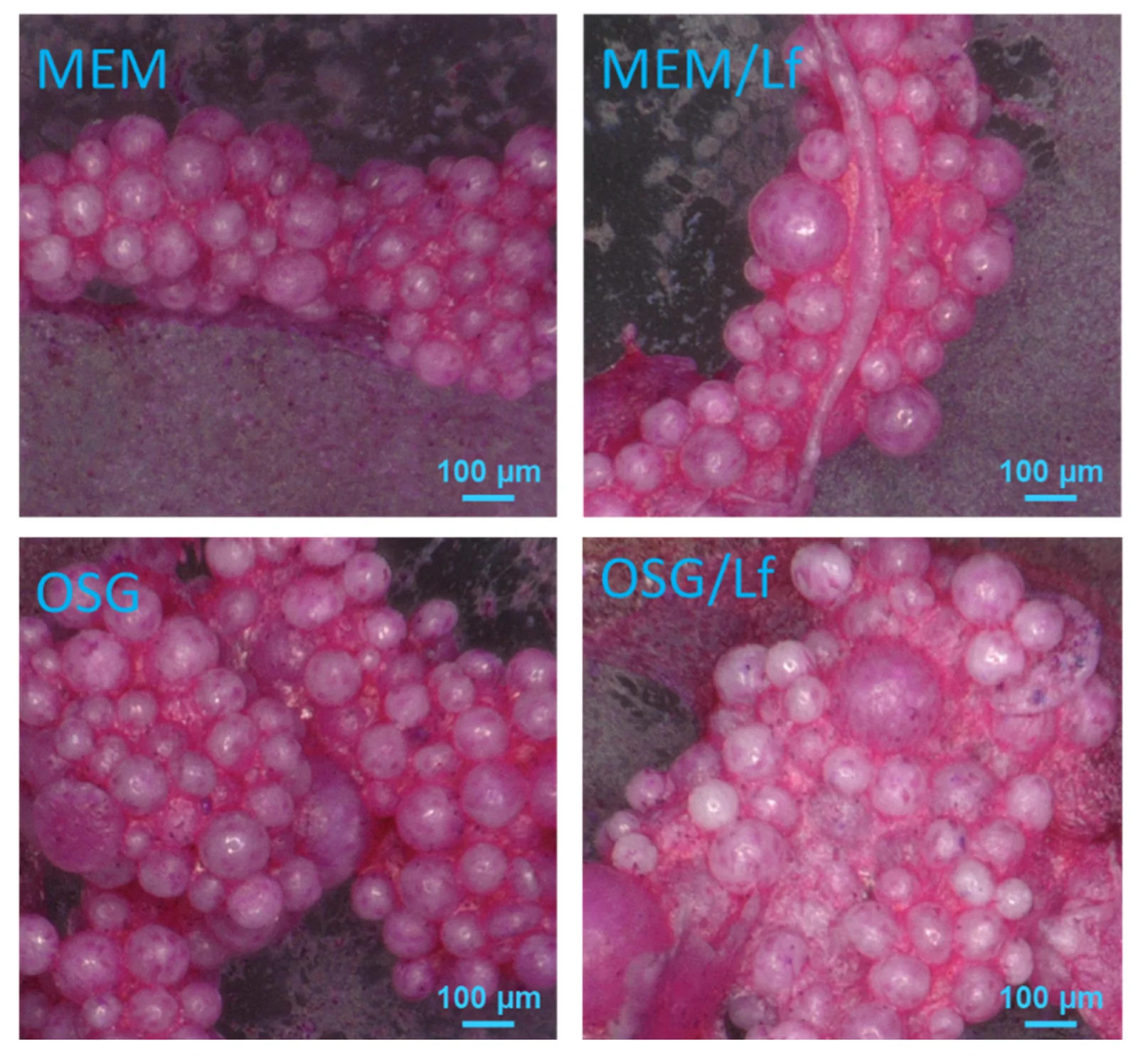
Microstructure of MSs after 14 days in contact with MC3T3 cells stained with hematoxin/eosin; MEM—MSs with minimal essential medium, MEM/Lf—MSs coated with Lf in minimal essential medium, OSG—MSs in OSG medium, OSG/Lf—MSs coated with Lf in OSG medium.

Optical microscopy using H/E staining Figure 10 confirms that the constructs, composed of numerous microspheres, are richly covered with cells. When compared to day 7, a notably higher density of cells is present within the core of the constructs, consistent with the SEM results. The morphology of the constructs is influenced by the culture medium: samples maintained in OSG medium form larger complexes of cells, ECM, and material, whereas those in MEM exhibit a more linear, elongated shape composed of a lower number of microspheres.

**Figure 11:**
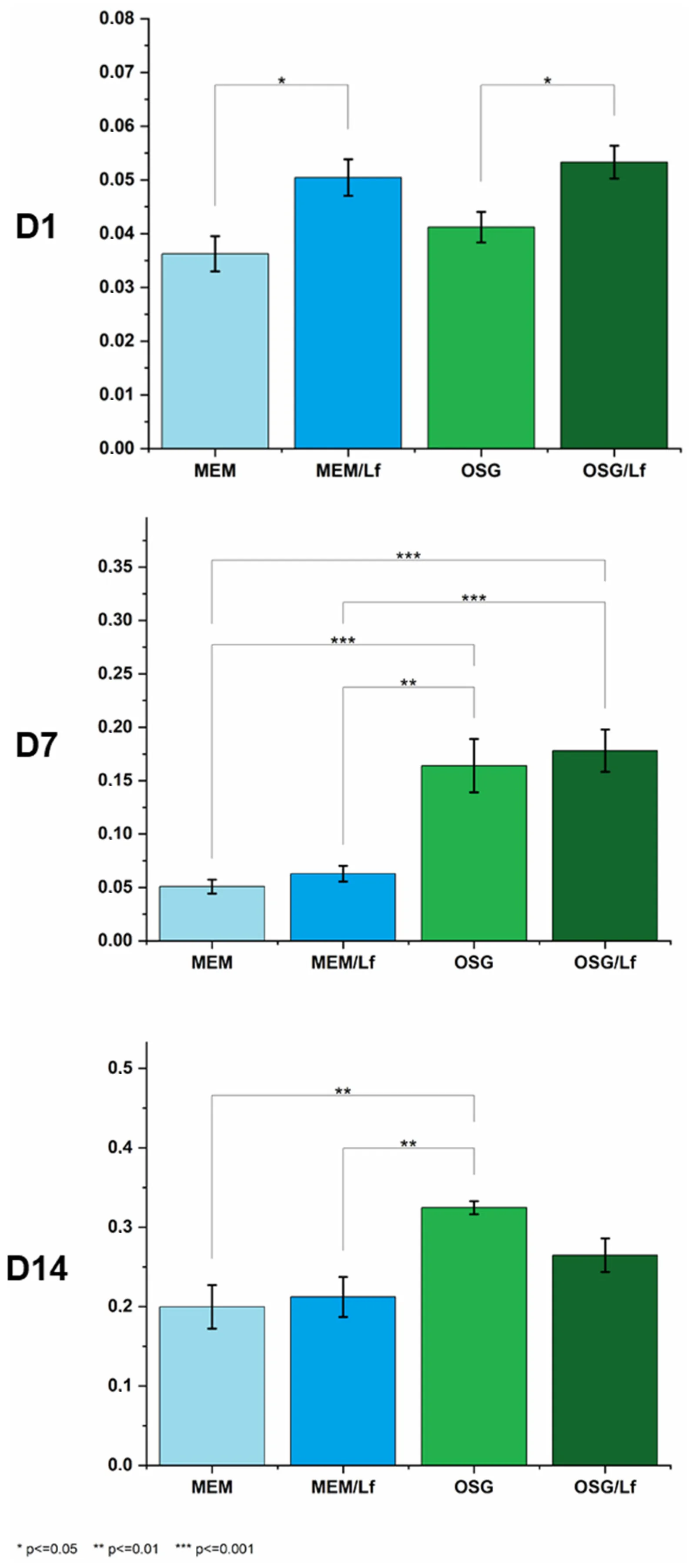
MTT cell viability test results after 1, 7 and 14 days in contact with MC3T3 cells; MEM—MSs with minimal essential medium, MEM/Lf—MSs coated with Lf in minimal essential medium, OSG—MSs in OSG medium, OSG/Lf—MSs coated with Lf in OSG medium.

The MTT cell viability test results comparing coated and uncoated microspheres (MSs) in MEM and OSG media are presented in Figure 11. At day 1, a clear trend is observed where the presence of Lf on the MS surface increases cell adhesion and proliferation, with a significant difference between coated and uncoated samples. There were no differences observed when comparing coated and uncoated samples across the different types of media. These initial 24 h indicate that Lf promotes cell adhesion to the material.

Day 7 shows a significant difference based on the type of medium used, with cell proliferation in OSG being approximately three times higher than in MEM. Cell proliferation increased for all samples compared to day 1. While proliferation was slightly higher for Lf-modified MS, this effect was too small to be statistically significant or classified as a positive effect of Lf at this time point.

Differences between samples at day 14 are considered relatively small. In MEM, there were no statistically significant differences, and a similar result was found in OSG, although proliferation was observably higher for MSs without Lf. By this day, the area of the MSs was almost completely covered, suggesting that observed differences may be due to the limited space available for cell growth. Ultimately, the primary positive effect of Lf on cell adhesion was observed at the initial time point.

**Figure 12:**
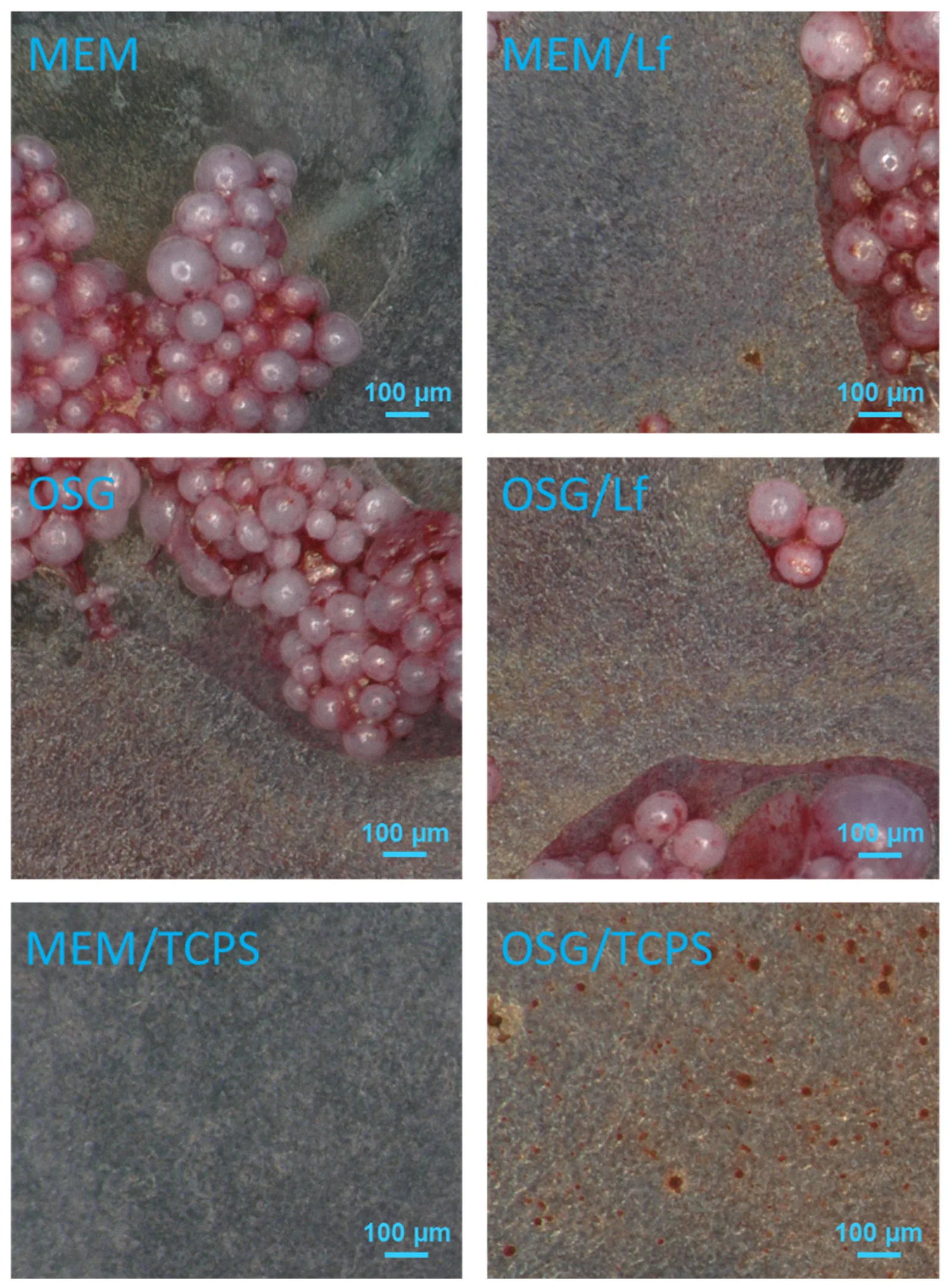
Alizarin Red staining of MSs after 14 days of growth of MC3T3 cells on their surface; MEM—MSs with minimal essential medium, MEM/Lf—MSs coated with Lf in minimal essential medium, OSG—MSs in OSG medium, OSG/Lf—MSs coated with Lf in OSG medium.

Alizarin Red staining (Figure 12) performed at day 14 provided evidence of osteogenic differentiation in MC3T3 cell cultures. As anticipated, cultures grown on tissue culture polystyrene (TCPS) in osteogenic (OSG) medium exhibited signs of differentiation into osteoblasts, characterized by the presence of reddish and goldish areas indicative of calcium ion deposition. In contrast, no differentiation was observed in MEM without differentiation supplements, as the color of the area remained unchanged.

The analysis of the microspheres (MSs) was primarily influenced by the inherent presence of hydroxyapatite (HAp) within the material’s volume and on its surface, which resulted in an intense red coloration on and around the microspheres. However, by focusing on the cells in the immediate vicinity of the materials, it was possible to assess differentiation. For MSs maintained in MEM, the results were consistent with expectations, showing no change in color and mirroring the MEM/TCPS control where cells did not differentiate. Conversely, in the sample of MSs coated with lactoferrin (Lf) in MEM, small areas of reddish and goldish color were observed, suggesting the presence of calcium ions and some degree of differentiation into osteoblasts, although these regions remained limited to the immediate neighborhood of the microspheres. Finally, for both MSs and MS/Lf samples cultured in OSG medium, calcium presence was detected with similar intensity across the entire surface of the well.

## 3. Discussion

The results of this study confirm that Lf is an effective surface modifier for PLA-based microspheres, creating a multifunctional material that promotes bone regeneration and differentiation while simultaneously addressing the risk of infection. The observed enhancement in cell adhesion and proliferation during the initial 24 h of culture is consistent with reports indicating that Lf, as a multifunctional glycoprotein, actively interacts with cell surface receptors, thereby facilitating initial attachment and promoting a pro-osteogenic environment [17,34]. Our findings regarding the “race for the surface”—a critical concept in biomaterial science—demonstrate that Lf-modified microspheres provide a significant competitive advantage to MC3T3-E1 cells over bacterial strains such as *P. aeruginosa* and *S. aureus* [26].

The ability of the microspheres to self-assemble into 3D constructs in the presence of osteoblasts is a notable observation. We observed that Lf coating significantly improves this process, resulting in larger, more integrated constructs. This suggests that Lf not only influences the cell–material interface but may also mediate cell–cell interactions through the extracellular matrix (ECM) proteins, as documented in previous studies on protein-mediated MS colonization [35]. The increased agglomeration observed in our experiments aligns with the known role of lactoferrin in modulating inflammatory and regenerative pathways, which may encourage a more robust cellular coverage of the synthetic scaffold [36].

Regarding the osteogenic differentiation confirmed by Alizarin Red staining, the intensity of the signal in the vicinity of Lf-coated microspheres in MEM suggests that Lf may possess intrinsic osteoinductive properties, independent of supplemental osteogenic media. While studies have shown that hydroxyapatite itself is osteoconductive [37], the supplementary effect of Lf in promoting calcium deposition suggests a synergistic interaction that warrants further investigation at the molecular level, particularly regarding the signaling pathways involved in osteoblast maturation. Regarding the antimicrobial profile, the comparatively limited efficacy of Lf against *C. albicans*—in contrast to its robust antibacterial performance—likely stems from fundamental differences in cell wall architecture. As highlighted in the recent literature, the mechanisms required for fungal inhibition differ significantly from those targeting bacterial membranes, often rendering Lf-based coatings less effective against fungal pathogens without additional synergistic agents [38].

A comparative analysis of the results obtained from the planktonic assay (Figure 5) and the surface adhesion assay (Figure 6) reveals interesting insights into the mode-dependent activity of the tested materials. While Figure 5 presents absolute CFU values in the surrounding medium, Figure 6 evaluates bacterial reduction relative to an untreated control, integrating both survival and surface colonization. Notably, unmodified microspheres already exhibit a substantial baseline antibacterial effect, which can lead to a “floor effect” where incremental improvements appear modest in absolute counts (Figure 5) yet become more pronounced when normalized against controls (Figure 6). Furthermore, lactoferrin immobilized on the microsphere surface exerts a strong localized effect at the material interface, whereas its availability in the surrounding planktonic medium is restricted. Consequently, microorganisms such as *S. aureus* exhibit distinct susceptibility profiles depending on whether they exist in a planktonic state or as surface-associated communities, highlighting the complementary nature of these evaluation methods.

While the present study provides a thorough evaluation of the antimicrobial efficacy and osteogenic biocompatibility of the Lf-modified microspheres, certain methodological limitations must be acknowledged. In biomaterials research, assessing antimicrobial properties and cytocompatibility through separate, well-controlled assays remains the established standard to reliably isolate each biological effect. Although a direct co-culture model involving both microbes and mammalian cells (such as MC3T3-E1) represents a compelling physiological approach to study the competitive “race for the surface,” it introduces significant experimental challenges. Incompatible culture media requirements, rapid microbial overgrowth over mammalian cells within hours, and critical pH shifts frequently obscure specific cellular responses. Consequently, to establish a foundational proof-of-concept, we intentionally utilized isolated assays. Building upon the strong anti-biofilm and osteogenic properties demonstrated independently in this work, future studies will focus on transitioning toward advanced dynamic co-culture models and in vivo evaluations to fully validate these initial findings under complex physiological conditions.

## 4. Materials and Methods

### 4.1. Synthesis and Characterization of Microspheres

Microspheres (MSs) were fabricated using an oil-in-water (o/w) emulsification method. The aqueous phase consisted of a 1% (*w*/*v*) poly(vinyl alcohol) (PVA) solution (Mowiol^®^; Mw ≈ 31 kDa). The oil phase was prepared by dissolving poly(L-lactic acid) (PLA, 3001D, NatureWorks, Plymouth, MN, USA) in dichloromethane (DCM) to achieve a 10% (*w*/*v*) solution, supplemented with 20% (*w*/*w* relative to PLA) hydroxyapatite (HAp, 21223, Sigma-Aldrich, St. Louis, MO, USA). Subsequently, 2 mL of the oil phase was added to 50 mL of the aqueous phase under constant magnetic stirring at 1000 rpm. The emulsion was stirred overnight to ensure complete evaporation of the DCM. The resulting MSs were collected, washed multiple times with distilled water, manually separated from any extraneous fibers or material residues, and air-dried.

The MSs were sterilized by immersion in 70% ethanol and dried within a laminar flow chamber. A subset of the microspheres was surface-coated with Lf under sterile conditions by adding 1.5 g of MSs to 50 mL of an aqueous lactoferrin solution (100 µg/mL), sterilized using a syringe filter (human lactoferrin, L1294, Sigma-Aldrich), and the mixture was left for 24 h.

Following incubation, the MSs were gently washed and dried in a laminar flow chamber.

The size and microstructure of the MSs were analyzed using a Keyence digital microscope (Osaka, Japan) and a NovaNanoSEM 200, FEI (Columbia, MO, USA) scanning electron microscope. Particle diameters were determined by measuring 500 microspheres; for each, two perpendicular measurements were taken, and the mean value was used to generate a size distribution histogram. Prior to SEM analysis, the samples were sputter-coated with a thin carbon layer to ensure conductivity. The morphological characteristics of the neat microspheres were analyzed using SEM at an accelerating voltage of 10 kV and a magnification of 2000×.

### 4.2. Microbiological Evaluation

To evaluate the antimicrobial efficacy of Lf-coated MSs, three reference strains from the American Type Culture Collection (ATCC) were utilized: *Staphylococcus aureus* (ATCC 25923), *Pseudomonas aeruginosa* (ATCC 2562) and *Candida albicans* (ATCC 90028).

The strains were stored at −80 °C in Tryptic Soy Broth (TSB) supplemented with 15% (*v*/*v*) glycerol. Prior to each experimental cycle, the strains were subcultured under the following conditions: *S. aureus* and *P. aeruginosa*: grown on Tryptic Soy Agar (TSA; Biomaxima, Lublin, Poland) at 37 °C for 24 h under aerobic conditions. *C. albicans*: grown on Sabouraud Dextrose Agar (Biomaxima, Lublin, Poland) at 37 °C for 48 h under aerobic conditions.

### 4.3. Quantitative Assessment of Microbial Adhesion

Microbial suspensions were prepared from fresh overnight cultures of the selected strains and adjusted to an optical density corresponding to 0.5 McFarland standard, approximately 1.5 × 10^8^ CFU/mL for bacteria and 1.5 × 10^6^ CFU/mL for fungi. Tryptic Soy Broth (TSB; Biomaxima, Lublin, Poland) and Sabouraud Dextrose Broth (SDB; Biomaxima, Lublin, Poland) were used as the culture media. The standardized suspensions were aliquoted into titration plates (FL Medical, Equimed, Krakow, Poland), and 100 µL of the material (0.1 g/mL in the respective broth) was added to each well. Following incubation (37 °C, 24 h, aerobic conditions, 100 rpm), the microspheres (MSs) were rinsed twice with 0.9% NaCl to remove non-adherent cells. The MSs were then transferred to Eppendorf tubes containing 1 mL of 0.5% saponin solution (Sigma-Aldrich, St. Louis, MO, USA) and vortexed for 1 min to detach the biofilm/adherent cells. The resulting suspensions were serially diluted (10^−1^–10^−6^) and quantitatively plated on Tryptic Soy Agar (TSA) for bacteria and Sabouraud Dextrose Agar for fungi. After incubation (37 °C, 24 h, aerobic conditions), the colonies were counted, and the number of colony-forming units per milliliter (CFU/mL) was determined. The CFU/mL value was calculated using the following formula: CFU/mL = average number of colonies × Dilution factor × 10. All experiments were performed in duplicate.

### 4.4. SEM Sample Preparation for Microbial Analysis

To prepare samples for Scanning Electron Microscopy (SEM), the MSs were rinsed once with 0.9% NaCl post-incubation. Samples were fixed with 4% paraformaldehyde aqueous solution for 15 min. Subsequently, the samples underwent dehydration through a graded ethanol series (40%, 50%, 60%, 70%, 80%, 90%, and 100%). Each step was performed for 10 min, followed by air-drying.

### 4.5. Antimicrobial Activity Against Planktonic Microbial Forms

The antimicrobial activity of the tested material against planktonic forms of the selected microorganisms was evaluated using the following protocol. Microbial suspensions were prepared from fresh overnight cultures and adjusted to an optical density corresponding to 0.5 McFarland standard (1.5 × 10^8^ CFU/mL for bacteria and 1.5 × 10^6^ CFU/mL for fungi). Tryptic Soy Broth (TSB) and Sabouraud Dextrose Broth (SDB) were used as the culture media. The standardized suspensions were aliquoted into titration plates (FL Medical), and 100 µL of the material (0.1 g/mL in the respective broth) was added to each well.

After incubation (37 °C, 24h, aerobic conditions, 100 rpm shaking), the suspensions were serially diluted and quantitatively plated onto Tryptic Soy Agar (TSA) for bacteria and Sabouraud Dextrose Agar for fungi. After a subsequent incubation period (37 °C, 24 h, aerobic conditions), colony-forming units were enumerated to determine the CFU/mL. All experiments were performed in duplicate, with microbial cultures incubated in the absence of the test material serving as negative controls.

### 4.6. In Vitro Assessment of Microspheres

#### 4.6.1. Cell Culture Conditions

Cytocompatibility, adhesion, and proliferation assays were performed using the MC3T3-E1 pre-osteoblast cell line (ATCC, Subclone 4, CRL-2593) with eight replicates (n = 8) for each experimental condition. MC3T3-E1 cells (ATCC) were retrieved from liquid nitrogen storage. A cryovial at passage 4 was thawed slowly, and the contents were gently mixed with 4 mL of supplemented Minimum Essential Medium (MEM). After 24 h of incubation, the culture medium was replaced. The cells were maintained until reaching approximately 80% confluence, at which point they were subcultured into two new culture flasks. Upon reaching ~80% confluence again, the cells were detached, pooled together, and prepared for the experiments.

Two types of microspheres (PLA/HAp and Lf-coated PLA/HAp) were evaluated in two different culture media: (I) Minimum Essential Medium (MEM, Gibco, Waltham, MA, USA) supplemented with 10% Fetal Bovine Serum (FBS) and 1% penicillin/streptomycin (Sigma-Aldrich); and (II) Osteogenic Medium (OSG), consisting of the same base composition supplemented with ascorbic acid (0.2 mM, Sigma-Aldrich) and β-glycerophosphate (10 mM, Sigma-Aldrich).

MSs were suspended in the appropriate medium (10 mg/mL), and 100 µL of the suspension was added to each well of a 96-well plate designed for suspension cultures (Sarstedt). Subsequently, 100 µL of medium (MEM or OSG) containing 5 × 10^3^ MC3T3 cells was added to each well. Cells were maintained in a humidified incubator at 37 °C in a 5% CO_2_ atmosphere. The culture medium was refreshed every 3–4 days (on days 3, 7, and 10).

#### 4.6.2. Cell Viability Assay (MTT)

Cell viability was assessed on days 1, 7, and 14 using the MTT assay, following the ISO 10993-5 standard [39]. At each time point, the culture medium was removed, and the wells were washed with Phosphate-Buffered Saline (PBS, pH 7.4, Gibco). Subsequently, 100 µL of MTT solution (1 mg/mL; Thiazolyl Blue Tetrazolium Bromide, Sigma-Aldrich) was added to each well, followed by incubation for 2 h at 37 °C. After removing the MTT solution, 100 µL of isopropanol was added to dissolve the formazan crystals. The plate was shaken gently for 30 min, and the absorbance was measured at 570 nm using an Epoch microplate reader (BioTek, Winooski, VT, USA).

#### 4.6.3. Osteogenic Differentiation (Alizarin Red Staining)

On day 14, mineralization was evaluated using Alizarin Red staining. The culture medium was removed, and the samples were washed with PBS, followed by fixation in ice-cold 70% ethanol for 1 h at −20 °C. After air-drying, the samples were incubated with Alizarin Red solution (Sigma-Aldrich) for 15 min at room temperature. The samples were then rinsed with deionized water and observed using optical microscopy to detect calcium deposits.

#### 4.6.4. SEM Sample Preparation

Samples for SEM analysis were prepared according to the protocol described in Section 4.4, with a slight modification: instead of rinsing with 0.9% NaCl, samples were washed with PBS prior to fixation in 4% paraformaldehyde. For the microbiological and MC3T3-E1 cell adhesion evaluations, SEM imaging was performed at an accelerating voltage of 10 kV and a magnification of 1000× to observe cell–material and microbe–material interactions.

### 4.7. Statistical Analysis

Statistical evaluations were performed using OriginPro (version 9.7.0.188) employing the Paired Comparison Plot App and the Tukey mean comparison method.

## 5. Conclusions

The results of this study demonstrate that microspheres can be effectively modified with lactoferrin to create a multifunctional biomaterial. The Lf-modified MSs exhibit significant antimicrobial activity, as evidenced by reduced bacterial adhesion and inhibited colony formation in *P. aeruginosa* and *S. aureus* suspensions. While the effect on *C. albicans* was limited, the overall reduction in bacterial proliferation underscores the potential of Lf as an antimicrobial agent.

Furthermore, the MSs supported the self-assembly of complex, three-dimensional constructs when cultured with MC3T3-E1 cells. Notably, the presence of the Lf coating enhanced this process, leading to larger constructs with a higher number of integrated microspheres, which suggests that Lf improves not only cell–material adhesion but also the stability of the cell-MS units themselves. The cell viability assays confirmed that Lf significantly increases initial cell adhesion to the material, providing a crucial advantage for host cells in the “race for the surface” against potential pathogens. The antibacterial properties of the Lf-coated MSs further bolster this competitive advantage in favor of the host cells.

Finally, Alizarin Red staining provided evidence of the osteogenic potential of the Lf-modified material. The detection of calcium-rich extracellular matrix (ECM) in samples cultured in MEM suggests that the Lf coating imparts an additional functionality that may facilitate osteogenesis and bone regeneration. In summary, lactoferrin serves as an effective multifunctional modifier for MSs. It confers antimicrobial properties; promotes MC3T3 cell adhesion, which is helpful in “the race for surface”; enhances the formation of 3D cellular constructs; and supports differentiation into osteoblasts. This unique combination of properties makes Lf-modified MSs a highly effective candidate for supporting bone tissue regeneration.

## Data Availability

The original contributions presented in this study are included in the article. Further inquiries can be directed to the corresponding author.
